# Geminated Supernumerary Premolar Impacted in the Palate: A Report of a Rare Case and Review of the Literature

**DOI:** 10.7759/cureus.46244

**Published:** 2023-09-30

**Authors:** Sayali K Desai, Omkar A Shetye, Rahul D Kamat, Pooja N Mandrekar, Rakshit V Sinai Khandeparker, Vikas Dhupar

**Affiliations:** 1 Oral and Maxillofacial Surgery, Goa Dental College and Hospital, Bambolim, IND

**Keywords:** radiography, cone beam computed tomography, impacted, gemination, supernumerary premolar

## Abstract

Supernumerary teeth are dental anomalies characterized by the presence of an excessive number of teeth in relation to normal dentition. Among these, the supernumerary premolars have a prevalence of 0.29% to 0.64%, making it a very rare finding. On the other hand, gemination is a developmental disturbance in the shape of the teeth where a partial cleavage of a single tooth germ results in the formation of a singular root and a singular pulp chamber but two partially or totally separated crowns. Although these anomalies as individual entities are fairly common in clinical practice, the occurrence of both anomalies in a single tooth is an extremely rare occurrence. We hereby report a rare case of impacted geminated supernumerary premolar in a 45-year-old female patient. A thorough search of the literature revealed that only four cases listing this anomaly have been reported in the literature thus deserving a mention. In addition to the case presentation, the authors have also reviewed the existing literature on this anomaly.

## Introduction

Supernumerary teeth represent the most common cause of odontostomatologic anomalies characterized by the presence of an excessive number of teeth in relation to the normal dentition occurring in the permanent dentition with a prevalence reported between 0.1% and 3.8% [1,2]. They may be unilateral, bilateral, single, or multiple involving either the maxilla or mandible. Despite the anterior maxilla being the most commonly involved region, the occurrence of supernumerary premolars has also been reported with a prevalence of 0.29% to 0.64%, making it a very rare finding [3]. On the other hand, gemination is a developmental disturbance in the shape of the teeth where a partial cleavage of a single tooth germ results in the formation of a singular root and a singular pulp chamber but two partially or totally separated crowns [4]. The reported prevalence in the permanent dentition is 0.1% for unilateral and between 0.02% and 0.05% for bilateral presentations, with the anterior maxilla being the most common site of presentation [5]. Although supernumerary teeth and gemination represent two separate odontostomatologic anomalies and are extremely rare, the occurrence of gemination in a supernumerary tooth present in the posterior maxilla (supernumerary premolar) is an extremely rare finding. A thorough search of the English literature on databases like PubMed, Scopus, and Embase revealed that only four such cases have been documented in the literature so far [6-9]. We hereby report a rare case of geminated supernumerary premolar impacted in the palate and discovered as an incidental radiographic finding in a 45-year-old female patient. An effort has also been made to review the existing literature about geminated supernumerary premolars.

## Case presentation

**Figure 1:**
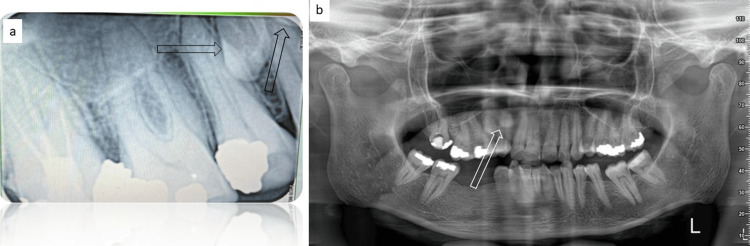
Conventional radiograph (IOPAR and OPG) views (a) Intraoral periapical radiograph (IOPAR) faintly showing the presence of a supernumerary tooth in relation to right maxillary premolars (black arrows). (b) Orthopantomogram (OPG) showing the presence of a supernumerary tooth with two crowns (white arrow).

A 45-year-old female patient of Asian ethnicity reported to our unit with pain in relation to the right maxillary second molar, which was previously managed endodontically. The patient had no significant medical history. The patient was subjected to an intraoral periapical radiograph (IOPAR), which revealed the presence of a supernumerary premolar in relation to the first and second premolars. Since the IOPAR was taken to visualize the second molar tooth, the supernumerary premolar was barely visible on the film; however, on close observation, it gave an impression that the supernumerary premolar had two separate crowns (Figure 1). An intra-oral clinical examination revealed a bulge in relation to the palatal aspect of the first and second premolars. No such bulges were observed in the rest of the quadrants, hence the patient was subjected to an orthopantomogram (OPG) as a screening tool to rule out the presence of additional supernumerary premolars in the remaining quadrants (Figure 1). The OPG revealed a radio-opacity in relation to the first and second premolars suggestive of a supernumerary premolar with two crowns. Such an occurrence was not noted in the other quadrants on the OPG. Syndromic association and familial tendency were also ruled out. The patient's chief complaint was addressed by the dentist by having the second molar retreated endodontically, following which the patient had no further episodes of pain in that tooth.

**Figure 2:**
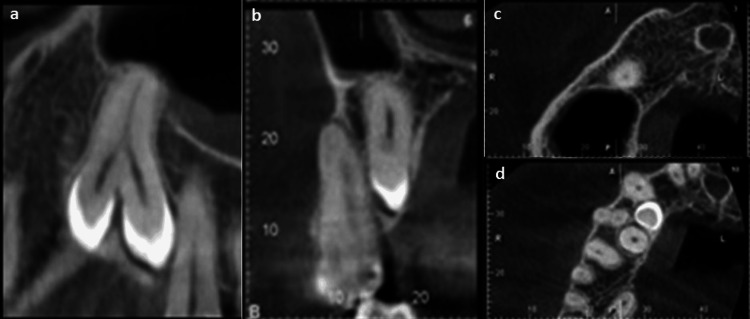
Cone-beam computed tomography with coronal, sagittal, and axial slices allowing for a three-dimensional study of the geminated supernumerary premolar tooth. (a) Coronal slice showing the morphology, root canal anatomy, and relationship of the tooth to the maxillary sinus. (b) Sagittal slice showing the impaction of the tooth in the palatal bone and its closeness to the first premolar root. (c) Axial slice showing the approximation of the root of the tooth to the maxillary sinus. (d) Axial slice demonstrating two crowns of the supernumerary premolar in close approximation to the roots of canine and first and second premolars.

To study the tooth in question in a detailed manner, a cone-beam computed tomography (CBCT) was advised for the patient, which confirmed the presence of an impacted supernumerary premolar with gemination of the tooth germ (Figure 2). The tooth measured 14.93 x 10.34 x 4.95 mm in greatest dimensions. Two crowns were seen erupting on the palatal aspect in the intra-radicular region of 13, 14, and 15 at the level of the alveolar crest. Mild contact was present between the crown present anteriorly and the middle third of the root of 13 while the rest of the tooth showed no contact with the surrounding teeth. Both the crowns were seen to fuse in the cervical third leading to a single root, which was seen extending up to the floor of the maxillary sinus with the apex of the root in close approximation with the right maxillary sinus lining. The maxillary sinus lining was seen to be thickened in relation to the floor of the maxillary sinus in proximity to the tooth root (Figure 2A). The tooth showed an intact pulp canal system with each crown having a single canal that merged at the cervical third leading to a single canal up to the apex. No periapical or pericoronal radiolucency was noted.

**Figure 3:**
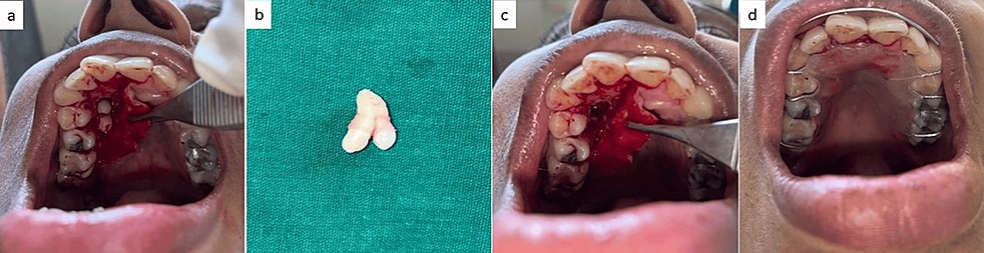
Intraoperative photographs of the patient (a) Flap reflection and exposure of the offending tooth. (b) Delivery of the tooth from the socket. (c) View of the surgical site following tooth removal. (d) Closure and placement of a palatal splint.

The risks and benefits of both treatment options, i.e., observation followed by periodic follow-up with OPG every year and surgical removal under either general or local anesthesia, and the associated risks were well explained to the patient. The patient was made well aware that with observation, there existed a 1-9.9% risk of cystic transformation in relation to the impacted supernumerary tooth [10]. Also, the risks of performing surgical removal of the offending tooth were explained to the patient, which included bleeding, oroantral communication, fracture of the palatal bone, damage to the adjacent tooth roots, infection, wound dehiscence, etc. Although the patient was asymptomatic with respect to the supernumerary premolar tooth, the patient opted to undergo surgical removal of the offending tooth under local anesthesia after thoroughly understanding both the treatment options that were put forward. An informed written consent was procured from the patient prior to the procedure. An alginate impression was taken of the maxillary arch and on the dental cast, a splint was fabricated with the purpose of eliminating the dead space and allowing for reattachment of the palatal mucosa back to its original position following the surgical procedure. Adequate anesthesia was secured using a combination of right greater palatine and nasopalatine nerve blocks. A crevicular incision was planned from the right central incisor to the right first molar using a number 15 surgical blade followed by a reflection of the full-thickness palatal mucoperiosteal flap, which allowed for visualization of the impacted supernumerary premolar. Bone removal, which acted as a hindrance to the smooth delivery of the tooth in question, was carried out using number 703 tapered fissure bur mounted on a straight handpiece under copious saline irrigation taking great care not to damage the tooth roots of the right maxillary canine and first and second premolars due to close proximity of the supernumerary premolar to these teeth (Figure 3). The tooth was then delivered out in toto, following which the integrity of the sinus lining was evaluated clinically by introducing a depth gauge used for the crestal sinus lift procedure (Hiossen, Englewood Cliffs, NJ) (Figure 3). The blunt end of the gauge made contact with the base, which revealed sound bone on all aspects, which ruled out an oroantral communication. The surgical wound was thoroughly irrigated with Betadine and normal saline and the flap was placed back in its original position and sutured using resorbable Vicryl sutures followed by placement of the fabricated surgical splint (Figure 3). As the sinus approximation was ruled out with the presence of sound bone following tooth removal, the patient was not advised of any sinus precautions. The patient was prescribed antibiotics (Augmentin 625 mg) for a period of five days and painkillers (ketorolac) for a period of four days post surgery. The healing proceeded uneventfully.

## Discussion

Although odontostomatologic anomalies concerning the shape and the number of teeth are fairly common in clinical practice; however, the occurrence of both anomalies in a single tooth is a rare phenomenon. This case report highlights the simultaneous occurrence of gemination in a supernumerary premolar. An extensive search of English literature revealed four cases documented in the literature [6-9], with the first case reported by Liu et al. in 2007 [6] in the mandibular premolar region, which resulted in the proposal of a new morphologic class “geminated premolar like” for the same. The details of the different cases of geminated supernumerary premolars are presented in Table 1.

**Table 1:** Documented cases of geminated supernumerary premolar/s reported in the literature CBCT: cone-beam computed tomography; IOPAR: intra-oral periapical radiograph; MDCT: multi-detector computed tomography.

Case	Author	Age/gender	Site (maxilla/mandible)	Unilateral/bilateral	Single/multiple	Investigations
1	Liu et al. (2007) [6]	19 years, male	Mandible	Unilateral	Single	CBCT
2	Yang (2012) [7]	35 years, male	Maxilla	Unilateral	Single	CBCT
3	Ather et al. (2012) [8]	19 years, male	Maxilla	Unilateral	Single	IOPAR, panoramic radiograph, MDCT
4	Soin et al. (2015) [9]	23 years, male	Maxilla and mandible	Bilateral (maxilla), unilateral (mandible)	Multiple	IOPAR, panoramic radiograph
5	Our case	45 years, female	Maxilla	Unilateral	Single	IOPAR, panoramic radiograph, CBCT

Based on the review of the literature, it was seen that geminated supernumerary premolars presented at different ages; therefore, a particular age group could not be specifically applied to them. Furthermore, all of the previous four documented cases involved males. This is the first case of geminated supernumerary premolar to involve the female gender. With respect to the involvement of the maxilla or mandible, three cases including this case have involved the maxilla, one has involved the mandible and one has involved both the maxilla and the mandible. Unilateral presentation was seen in four cases, including our case, whereas bilateral representation was seen in the maxilla and unilateral representation was seen in the mandible in the same patient in the remaining case. While four cases had a single geminated supernumerary premolar, one case had multiple.

Most supernumerary teeth present no symptoms and are usually impacted [11]. Therefore, they are usually discovered as incidental findings on routine radiographs just like in this case. Conventional radiographs like IOPAR are first resorted to, which provide a clear image of the target area. However, given the smaller size of the film, there is a high propensity that such an anomaly may be easily missed out, which was the case with this case as well. As the patient in this case came with the chief complaint pertaining to the right maxillary second molar, the IOPAR film was oriented in the oral cavity keeping the target tooth in mind and as a result, the impacted supernumerary geminated premolar was almost missed out. The authors are of the opinion that this is usually the case with most of these cases resulting in under-reporting of this anomaly. Panoramic radiographs can be considered as initial screening radiographs, which help to identify if there is a presence of such anomaly across all the quadrants. Multiple supernumerary teeth can be associated with non-syndromic cases. The presence of supernumerary tooth associated with other craniofacial/dental abnormalities should need a keen evaluation for syndromic association. Multiple supernumerary teeth associated with syndromes include Gardner’s syndrome, cleidocranial dysplasia, Fabry-Anderson syndrome, and cleft lip and palate [2]. Although conventional radiographs provide adequate details, they fail to provide definitive information about the relationship of the supernumerary tooth with vital structures like the maxillary sinus, adjacent teeth, nerves, etc. Therefore, advanced imaging techniques like CT and CBCT scans are instrumental in the preoperative evaluation as they provide a three-dimensional view of the tooth/teeth and assist in surgical planning [12]. In our case, CBCT provided valuable information regarding the intra-osseous position of the tooth whether buccal or palatal, its exact morphology and dimensions, as well as its proximity to the adjacent teeth, maxillary sinus, and cortical bone. Multidetector CT scans have also been employed for detection; however, given the higher radiation dose that the patient is subjected to, a CBCT is a better alternative. Based on the review of the literature of the existing and present cases, CBCT scanning was performed in three out of the five cases, including our case [6,7], whereas MDCT was performed in one case [8]. In the remaining case, although CBCT was advised, it was not performed due to financial constraints [9].

Management of such an anomaly is either by surgical removal or observation with periodic follow-up. Surgery for removal of such an anomaly can be performed in cases of cystic transformation, root resorption of adjacent teeth, pain or infection, or cases where there can be rotation or malformation, or delayed eruption of the permanent teeth [13]. However, if the impacted supernumerary geminated premolar is devoid of any periapical/pericoronal radiolucency or exhibits close proximity to vital structures like nerves or maxillary sinus such that removal can lead to nerve damage or an oroantral communication, the tooth/teeth can be retained and observed periodically. Soin et al. [9] opine that the abnormal anatomy of the anomaly contributes to complications and poor prognosis and therefore advocate the removal of such teeth. In our case, the patient after understanding both the treatment options opted for tooth removal despite it being asymptomatic and despite it being in close proximity to the maxillary sinus. We did not encounter an oroantral communication, which was confirmed after the tooth removal, and the healing was seen to progress uneventfully. In the event of an oroantral communication in such a case, emphasis should be placed on achieving a water-tight mucosal closure, application of the splint with adequate sinus precautions consisting of avoidance of sneezing and nose blowing for at least two weeks, and use of xylometazoline nasal drops and steam inhalation to relieve the nasal stuffiness, which would allow for adequate management without having to resort to the use of either local or free soft tissue flaps.

## Conclusions

An impacted supernumerary geminated premolar represents a rare dental anomaly that deserves a mention in the literature. Although conventional radiography has a limited role in preoperative evaluation and surgical planning, nevertheless, it is important to establish if the occurrence is single or multiple, involving one or both jaws. The role of CBCT is instrumental in both the preoperative evaluation and treatment planning so that surgical removal can be carried out in the best possible way. The decision to either wait and watch or surgically intervene should be on an individual case basis, taking into consideration the risks and benefits of either treatment option.

## References

[1] Rajab LD, Hamdan MA (2002). Supernumerary teeth: review of the literature and a survey of 152 cases. Int J Paediatr Dent.

[2] Subasioglu A, Savas S, Kucukyilmaz E, Kesim S, Yagci A, Dundar M (2015). Genetic background of supernumerary teeth. Eur J Dent.

[3] Kasat VO, Saluja H, Kalburge JV, Kini Y, Nikam A, Laddha R (2012). Multiple bilateral supernumerary mandibular premolars in a non-syndromic patient with associated orthokeratised odontogenic cyst- a case report and review of literature. Contemp Clin Dent.

[4] Aryanpour S, Bercy P, Van Nieuwenhuysen JP (2002). Endodontic and periodontal treatments of a geminated mandibular first premolar. Int Endod J.

[5] Mahendra L, Govindarajan S, Jayanandan M, Shamsudeen SM, Kumar N, Madasamy R (2014). Complete bilateral gemination of maxillary incisors with separate root canals. Case Rep Dent.

[6] Liu DG, Zhang WL, Zhang ZY, Wu YT, Ma XC (2007). Three-dimensional evaluations of supernumerary teeth using cone-beam computed tomography for 487 cases. Oral Surg Oral Med Oral Pathol Oral Radiol Endod.

[7] Yang G (2012). Supernumerary teeth and gemination. Br J Oral Maxillofac Surg.

[8] Ather A, Ather H, Sheth SM, Muliya VS (2012). Unique case of a geminated supernumerary tooth with trifid crown. Imaging Sci Dent.

[9] Soin A, Sharma G, Soin G, Raina A, Mutneja P, Nagpal A (2015). Multiple geminated supernumerary premolars: a rare case report. Case Rep Dent.

[10] Jiang Q, Xu GZ, Yang C, Yu CQ, He DM, Zhang ZY (2011). Dentigerous cysts associated with impacted supernumerary teeth in the anterior maxilla. Exp Ther Med.

[11] Açikgöz A, Açikgöz G, Tunga U, Otan F (2006). Characteristics and prevalence of non-syndrome multiple supernumerary teeth: a retrospective study. Dentomaxillofac Radiol.

[12] Kim KD, Ruprecht A, Jeon KJ, Park CS (2003). Personal computer-based three-dimensional computed tomographic images of the teeth for evaluating supernumerary or ectopically impacted teeth. Angle Orthod.

[13] Anthonappa RP, Omer RS, King NM (2008). Characteristics of 283 supernumerary teeth in southern Chinese children. Oral Surg Oral Med Oral Pathol Oral Radiol Endod.

